# Publisher Correction: Cryo-EM structures reveal how phosphate release from Arp3 weakens actin filament branches formed by Arp2/3 complex

**DOI:** 10.1038/s41467-024-46804-9

**Published:** 2024-03-15

**Authors:** Sai Shashank Chavali, Steven Z. Chou, Wenxiang Cao, Thomas D. Pollard, Enrique M. De La Cruz, Charles V. Sindelar

**Affiliations:** 1 Department of Molecular Biophysics and Biochemistry, Yale University, PO Box 208103, New Haven, CT 06520-8103 USA; 2 Department of Molecular Cellular and Developmental Biology, Yale University, PO Box 208103, New Haven, CT 06520-8103 USA; 3 Department of Cell Biology, Yale University, PO Box 208103, New Haven, CT 06520-8103 USA; 4 Department of Molecular and Cell Biology, University of California, 638 Barker Hall, Berkeley, CA 94720-3200 USA; 5 Present Address: Department of Molecular Biology and Biophysics, University of Connecticut Health Center, Farmington, CT 06030 USA

**Keywords:** Cryoelectron microscopy, Cellular motility, Cytoskeletal proteins

Correction to: *Nature Communications* 10.1038/s41467-024-46179-x, published online 06 March 2024

In this article the ‘Results’ heading was inadvertently captured as part of the subsequent subheading ‘Cryo-EM structure of a mature Arp2/3 complex branch junction at 2.7 Å resolution’. The original article has been corrected.

