# Supplementary material for: Cryo-EM structures reveal how phosphate release from Arp3 weakens actin filament branches formed by Arp2/3 complex
Source: Nat Commun. 2024 Mar 6;15:2059. doi: 10.1038/s41467-024-46179-x (PMC10918085; doi:10.1038/s41467-024-46179-x)
Supplement: Supplementary file 1 — Supplementary Information [file 41467_2024_46179_MOESM1_ESM.pdf]

Cryo-EM structures reveal how phosphate release from Arp3 weakens actin filament branches formed by Arp2/3 complex

Sai Shashank Chavali[1], Steven Z. Chou[2,#], Wenxiang Cao[1], *Thomas D. Pollard[1,2,3,4], *Enrique M. De La Cruz[1], and *Charles V. Sindelar[1]

[1]Department of Molecular Biophysics and Biochemistry

[2]Department of Molecular Cellular and Developmental Biology

[3]Department of Cell Biology

Yale University, PO Box 208103, New Haven, CT 06520-8103 USA

[4]Department of Molecular and Cell Biology, University of California, Berkeley

#Current address: Department of Molecular Biology and Biophysics, University of Connecticut Health Center, Farmington CT 06030

*Correspondence: Address correspondence to thomas.pollard@yale.edu, enrique.delacruz@yale.edu, or charles.sindelar@yale.edu

Supplementary Information

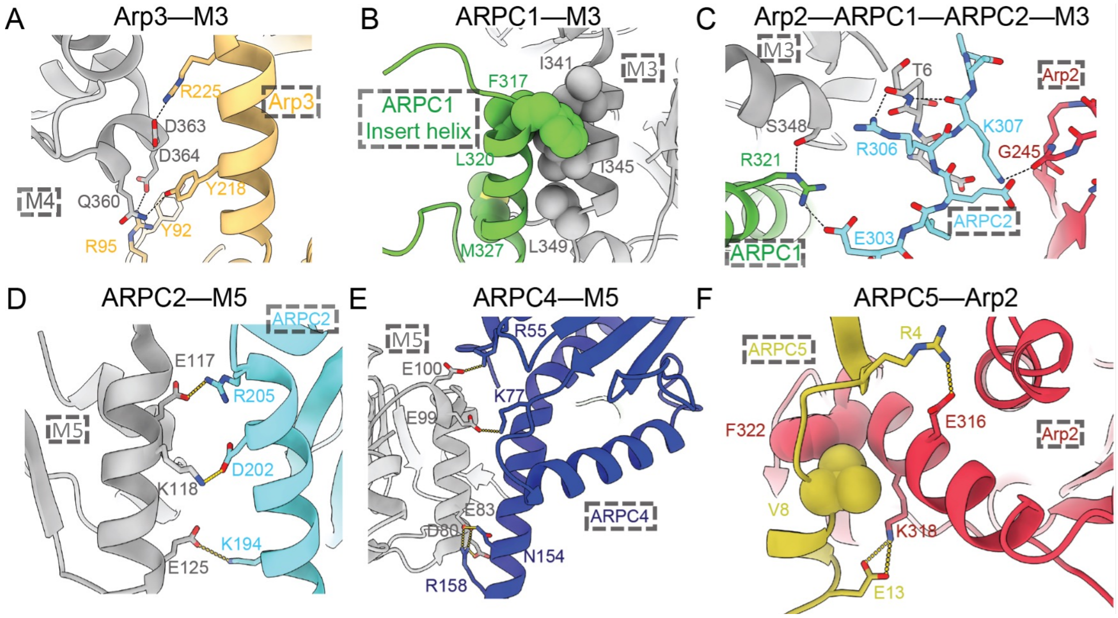

Supplementary Fig. 1: Specific interactions observed between Arp2/3 complex and mother filament:

A, Arp3 interacts with M3 actin subunit through salt bridge interactions. Both R95 and R225 make salt bridge interactions with neighboring D364 and D363 residues respectively. Y218 makes an additional hydrogen-bond interaction with Q360.

B, The hydrophobic interface of the ARPC1 insert helix (F317, L320, and M327) with actin subunit M3 (I341, I345, and L349). The space filling spheres represent van der Waals radii that reflect potential contact points between the hydrophobic residues. An additional hydrophobic contact occurs between M327 (ARPC1) and the D-loop of the M4 actin subunit (M47; not shown).

C, ARPC2 (cyan) bridges a large interface comprising ARPC1 insert helix (green), Arp2 (red) and actin subunit M3 (grey). R306 in ARPC2 forms a hydrogen-bond with the N-terminus residue T6 of M3 actin subunit. K307 on the other hand makes a hydrogen-bond interaction with the backbone of G245 in Arp2. Another network of salt bridge and hydrogen bond interactions between R321in the ARPC1 insert helix and E303 in the ARPC2 C-terminal helix and side chain of S348 in SD1 of M3 actin subunit stabilizes the large interface spanning ARPC1, ARPC2, Arp2 and actin M3.

D, ARPC2 also interacts with the mother filament subunit M5 through salt bridges between K194, D202 and R205 in ARPC2 and E125, K118 and E117 of M5 subunit respectively.

E, ARPC4 makes strong contacts with SD4 of M5 actin subunit by engaging R55 and K77 in salt bridges with negatively charged residues E99 and E100 in M5 subunit. In addition, R158 contacts two negatively charged residues D80 and E83 simultaneously through salt bridges.

F, ARPC5 does not make specific interactions with the mother filament but utilizes its N-terminal residues to bind SD3 of Arp2, thus stabilizing its interface with ARPC4. Salt bridges are formed between R4 and E13 in ARPC5 and E316 and K318 in Arp2 respectively. A hydrophobic interfaces comprising V8 in ARPC5 and F322 in Arp2 contribute to the stability. Space filling models represent contact points between the hydrophobic residues.

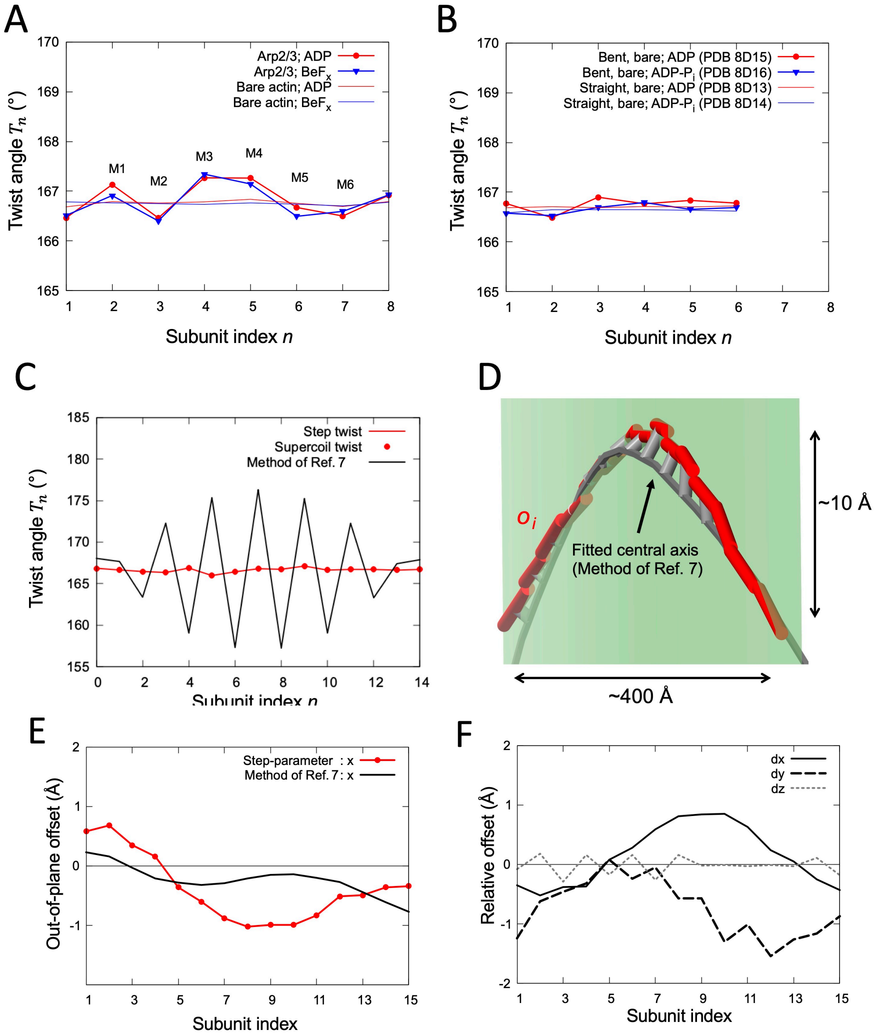

Supplementary Fig. 2. Measurement of twist perturbations in actin filaments by three methods. The 'step-parameter' formula of Britton et al.[1] is one of several related methods used to measure twisting in DNA structures [2-6]. The step-parameter twist is obtained by estimating subunit orientations directly from fit subunit atomic coordinates (Fig. 2A); the result is independent of shearing between subunits and other irregularities in the filament path[1]. A second twist measure described by Britton et al., 'supercoil twist', is closely related and gives similar results; however, if filaments are strongly curved the supercoil twist becomes sensitive to shearing and other irregularities [1]. A third method, introduced by reference [7], is based on a geometric analysis of a spline curve that follows the subunit centers-of mass.

A, Inter-subunit twisting in the mother filament caused by binding of Arp2/3 complex for ADP (red) and ADP-BeF$_x$ (blue) samples. Plot of estimated step-parameter twist angles between subunits i and i+1 for control bare actin filaments (lines) and mother filaments (lines/symbols) in Arp2/3 complex branch junctions. Bare filament measurements were obtained from samples with branches reconstructed without symmetry. The +/- 0.5° deviations in twist along the mother filament represent structural deformations due to Arp2/3 complex binding; in comparison, deviations in the control (bare-filament) twist are much smaller (mean and standard deviation are

-166.76° +/- 0.02°, respectively for the ADP sample; and -166.74° +/- 0.037°, respectively for the BeF_x sample) indicating that Arp2/3 binding has caused significant (> 10 $\boldsymbol{\sigma}$) inter-subunit twisting deviations in the mother filament, compared to the twist deviations in bare actin.

B, Step-parameter twist estimates (lines/symbols) for curved, bare actin filament structures (PDB ID's 8D16 [https://doi.org/10.2210/pdb8D16/pdb] and 8D15 [https://doi.org/10.2210/pdb8D15/pdb] for ADP-P_i and ADP actin, respectively [7]). Control measurements (lines) were made using actin subunit coordinates individually fit in deposited straight-filament, asymmetric reconstructions from the same samples as the curved structures (EMD-27115 [https://doi.org/10.2210/pdb8D14/pdb], EMD-27114 [https://doi.org/10.2210/pdb8D13/pdb], respectively [7]). Inter-subunit twists deviate much less from the average than in branch complex structures. Means and standard deviations in the control (straight-filament) twist are -166.70° +/- 0.035°, respectively for the ADP sample; and -166.64° +/- 0.05°, respectively for the ADP-P_i sample)

C, Comparison of three methods to estimate filament twist of a bent, 15-subunit, ADP-P_i-actin filament (PDB model ADP_Pi_cryodrgn_isolde_frame009.pdb from the data supplements of reference [7]). The step-parameter and supercoil twist values (red line and red markers, respectively) are close to the symmetric value for actin (166.7 degrees) and indistinguishable from each other, differing by less than 0.002° for all subunit positions. In contrast, twists estimated by the method of reference [7] (black line) oscillate by up to +/-13° between neighboring subunits; moreover, these oscillations exhibit a low-frequency 'beat' phenomenon, where oscillations are periodically damped to near-zero amplitude every ~13 subunits.

D, Comparison of the path o_i calculated for bent, 15-subunit filament structure in panel C by the methods used here (red cylinders depict tangent vectors o_i centered at o_i as in Fig. 2C-G) vs. a path of the same structure estimated using the method of reference [7]. Our path estimate for the bent actin structure undulates back and forth between opposite sides of the bending plane, with a period of ~13 subunits and an amplitude of ~1 Å (see panels E, F below) . This period matches the helical pitch of actin filaments (13.5 subunits). These oscillations probably represent a short-range effect due to bending anisotropy inherent in the actin filament structure; such features are averaged out in macroscopic measurements involving longer length scales [8]. The path estimate by reference [7] (gray 3D curve) exhibits similar oscillations but is offset by up to 2 Å from our path offset. Displacements between corresponding path coordinates of the two methods are depicted using gray 3D cylinders.

E, Comparison of the filament path displacements for the two path estimates, as a function of subunit number, in the direction orthogonal to the bending plane. The displacements exhibit a similar oscillating character (reflecting the undulations through the bending plane), but the oscillations for the two methods are phase-shifted by ~4 subunits.

F, Plot of subunit-wise differences dx, dy, dz between filament path coordinates of the two methods, plotted as a function of subunit number. Due to these systematic, oscillating discrepancies, the filament paths of the two methods coincide only once every ~13 subunits, with maximum deviations exceeding 2 Å.

Source data for panels A-C, E-F are provided as a Source Data file.

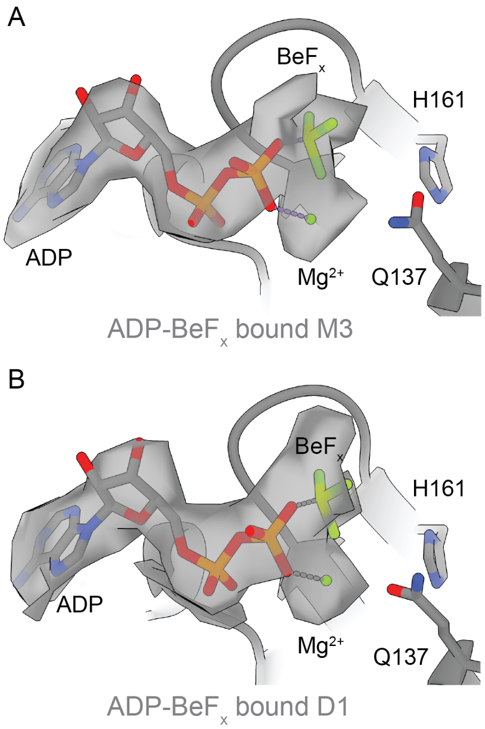

Supplementary Fig. 3: Evidence for BeF_x corresponding to the γ-phosphate position in mother and daughter actin subunits. A, Electrostatic potential density showing distinguishable evidence for BeF_x and Mg^{2+} in mother actin subunit M3. Nucleotide densities for mother subunits M1-M5 are mostly indistinguishable, while the density at the BeF_x site is weaker in subunit M6, likely due to lower resolution near the volume periphery. B, Density supporting BeF_x in the γ-phosphate position for daughter actin subunit D1.

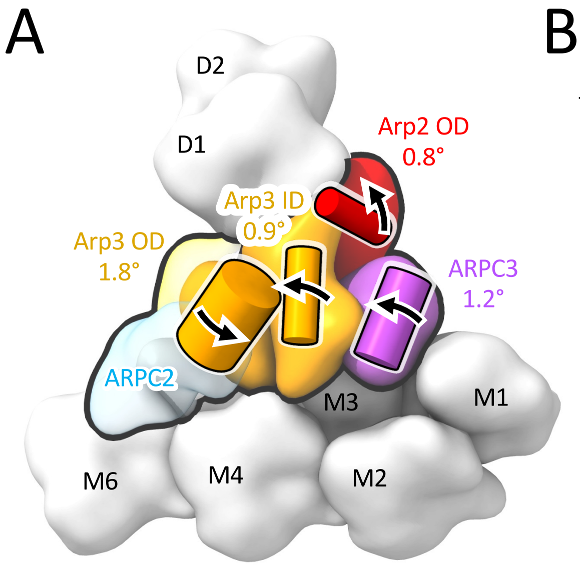

B

Daughter	Shift	Rot.
D2	0.5 Å	0.2°
D1	0.4 Å	0.3°

Anchor	Shift	Rot.
ARPC5	0.1 Å	0.4°
ARPC1	0.2 Å	0.5°
ARPC2	0.1 Å	0.3°
ARPC4	0.2 Å	0.2°

Hinge	Shift	Rot.
Arp2 ID	0.2 Å	0.3°
Arp2 OD	0.4 Å	0.9°
Arp3 OD	0.4 Å	1.8°
Arp3 ID	0.3 Å	1.0°
ARPC3	0.4 Å	0.8°

Mother	Shift	Rot.
M1	0.1 Å	0.5°
M2	0.2 Å	0.3°
M3	0.1 Å	0.2°
M4	0.1 Å	0.3°
M5	0.1 Å	0.3°
M6	0.2 Å	0.3°

Supplementary Fig. 4. Subunit rotations and translations in the branch junction structures, for the transition from ADP-BeF_x to ADP. A, 'Hinge' subunit rotations in Arp2/3 complex. Rendering, coloring and view are the same as Fig. 5A. Cylinders superimposed over Arp2, Arp3 and Arp C3 indicate the axis of rotation, and cylinder diameters are scaled proportional to the rotation magnitude. B, Tables of all subunit translations and rotations in the branch complex, organized by region (daughter filament, anchor, hinge, mother filament; see Fig. 5C). For both shifts and rotations, values fall into low and high groupings; high-valued groups are highlighted in pink.

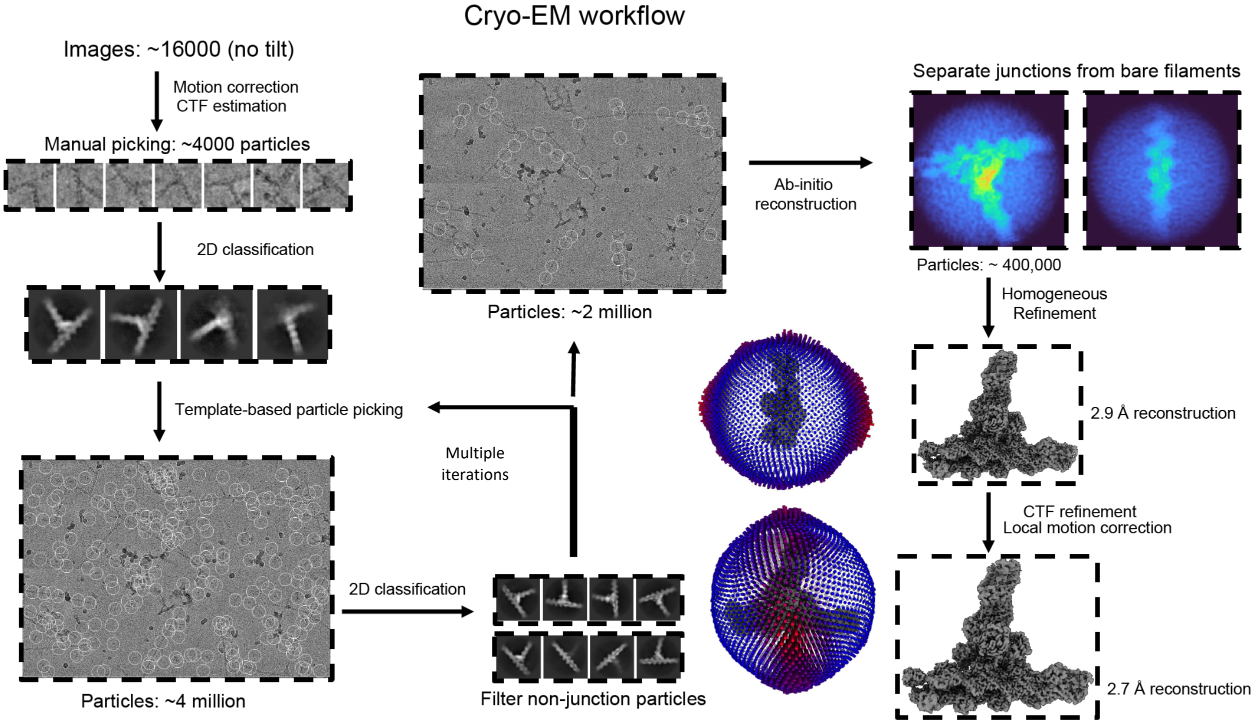

Supplementary Fig. 5: Cryo-EM workflow employed to pick branch junction particles and reconstruct high-resolution maps: ~16,000 images without tilt were collected for the mature (ADP) branch junction sample and ~8700 images (~4200 images without tilt and ~4500 images with a tilt of 20°) were collected for the ADP-BeF_x sample. Both datasets were processed the same way using cryoSPARC software [9]. About 4000 particles (from both ADP and ADP-BeF_x samples) that contained a "Y-shaped" branch junction were manually picked using a box size of 256 x 256 Å. These particles include rare views where the daughter filaments were parallel (nearly parallel) to the optical axis. Then, 2D classification was performed to create templates for template-based particle picking. The first round of template-based particle picking produced ~4 million ADP particles and ~2 million for ADP-BeF_x particles that included various non-junction, bare filament, and non-protein junk particles. Several rounds of 2D classification were then performed to filter out non-junction particles. The resulting particles, ~2 million (ADP state) and ~ 1.2 million (ADP-BeF_x state), included both junction particles but also bare actin filament particles. These particles were then subjected to ab-initio reconstruction (with 10 classes) to separate junction particles from bare filament particles. The final set of particles used for 3D reconstruction was ~400,000 for ADP state and ~200,000 for ADP-BeF_x state. A homogeneous reconstruction of these particles produced maps at 2.9 Å (ADP state) and 3.4 Å (ADP-BeF_x). CTF refinement and local motion correction was then performed to improve the resolution, that resulted in final reconstructions with resolutions of 2.7 Å (ADP) and 3.2 Å (ADP-BeF_x). The angular distribution diagrams indicate a uniform distribution of particles in each orientation (red: preferred orientation and blue: no preferred orientation).

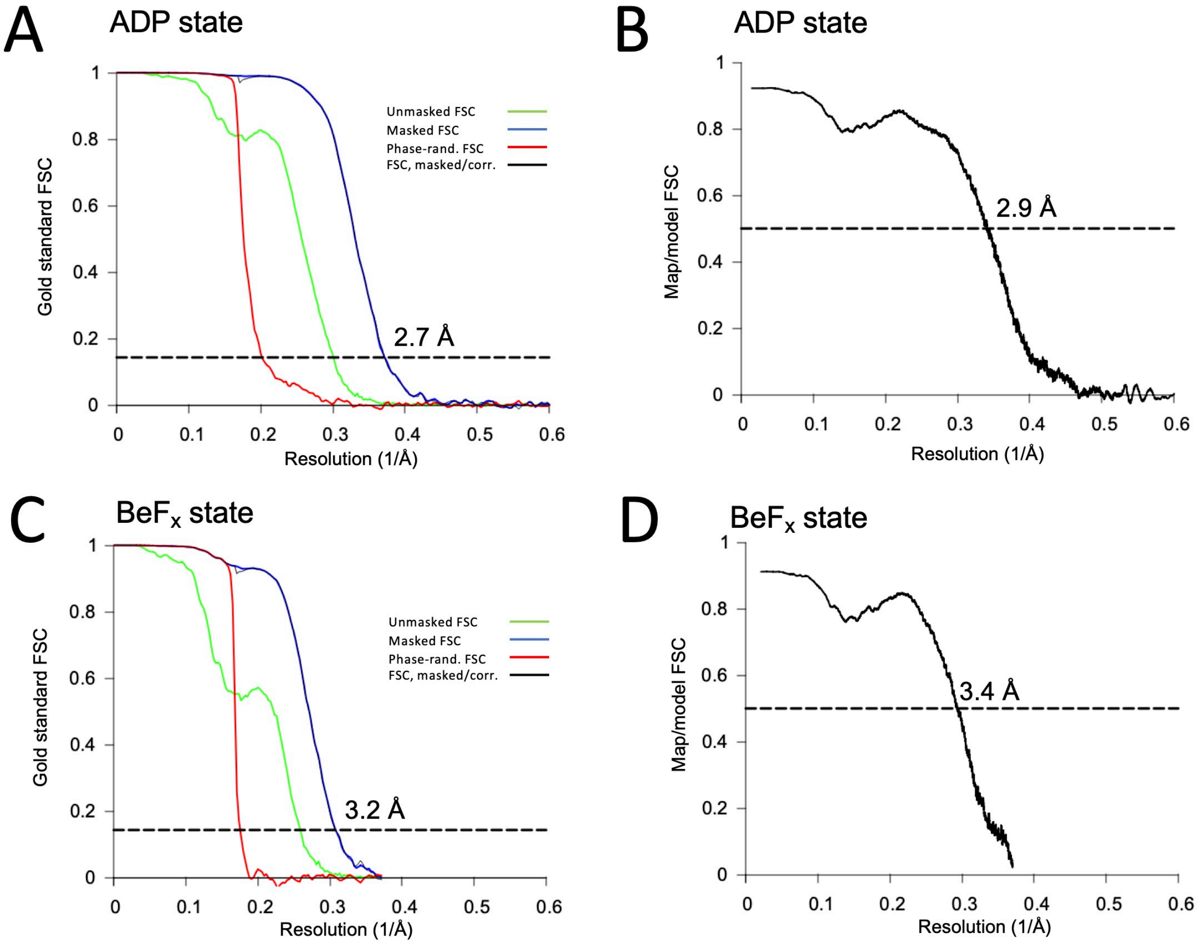

Supplementary Fig. 6: Resolution estimation of ADP and ADP-BeF_x bound branch junction structures illustrated by Fourier shell correlation plots. The 'relion_postprocess' command from the Relion software suite was used for FSC calculations. To reduce artifacts due to truncation of the actin filament at mask boundaries, 'tight' masks output by cryosparc refinement jobs were further softened by multiplying with a low-resolution, soft-edged mask generated from our branch atomic structures. For map-model FSC calculations, synthetic maps were generated from model atomic coordinates using UCSF ChimeraX ('molmap' command). The experimental map, synthetic map and mask (identical to the one used for half-map FSC calculations) were then input to the relion_postprocess program. A, Half-map FSC analysis for the ADP structure. B, Map-model FSC curve for the ADP structure. C, Half-map FSC analysis for the BeF_x structure. D. Map-model FSC curve for the BeF_x structure. Horizontal lines represent the 0.143 resolution cutoff criterion (half-map FSC) or 0.5 resolution cutoff criterion (map-model FSC). Source data are provided as a Source Data file.

Supplementary Notes

Net twist difference measurements of actin filament structures

The net twist difference [10] (excess end-to-end twist, Fig. 2J-K), here denoted $\Delta T_{i,n}$, was computed as the measured end-to-end twist angle between subunit *i* and *n* of a distorted actin filament segment ($T_{i,n} = T_i + T_{i+1} + \cdots + T_n$) minus the measured average end-to-end twist of a bare, straight reference actin filament ($\overline{T_{i,n}^{\mathrm{ref}}} = \overline{T_i^{\mathrm{ref}}} + \overline{T_{i+1}^{\mathrm{ref}}} + \cdots + \overline{T_n^{\mathrm{ref}}} = (n - i + 1)\overline{T_i^{\mathrm{ref}}}$), resulting in the expression $\Delta T_{i,n} = T_{i,n} - \overline{T_{i,n}^{\mathrm{ref}}}$. Here, a twist T_i with one subscript index is defined by the rotation angle for ***l*** vector rotating about tangent vector ***n*** from a filament subunit *i* to the next subunit *i* + 1 (Fig. 2A); we follow the formulas and conventions of Ref. [1] to compute either 'supercoil' or 'step-parameter' twist values, which give essentially identical results for the structures analyzed here (Fig. S2C).

Differences between actin twist analysis reported here and in Ref. [7]

The filament twist values of bent filaments reported here differ from those obtained by Ref. [7] (Fig. S2C). Both analyses rely on determining the filament trajectories from structural models obtained by electron cryomicroscopy. The methods for doing so, however, are distinct, and yield different results.

Filament path undulations identified in Fig. S2D-F provide a clue as to the origin of this difference. In contrast to the step-parameter methods to measure twist used here, twist estimates obtained by the methods used in reference [7] depend strongly on how the filament path is defined, which is ambiguous for non-ideal, experimentally determined structures [1]. Presumably, the large twist oscillations reported by reference [7] (Fig. S2C) reflect the choice of path. The filament path ('central axis') in reference [7] was estimated by a spline curve fit to filament subunit centers-of-mass using a customized, iterative algorithm, and this path deviates systematically from the path obtained by the methods used here (Fig. S2D-F).

Another potential source of the differences may originate from the use of the Frenet-Serret reference frame in reference [7]. Because torsion integrals in this reference frame diverge, the resulting quantities (such as twist and writhe) are also diverge [11]. Structural parameters estimated in this way can therefore become corrupted for polymers undergoing small, thermally-driven fluctuations in shape [11], such as the filaments analyzed here and in study [7] .

Supplementary References:

1. Britton, L.A., Olson, W.K. & Tobias, I. Two perspectives on the twist of DNA. *J Chem Phys* **131**, 245101 (2009).
2. Lu, X.J. & Olson, W.K. 3DNA: a software package for the analysis, rebuilding and visualization of three-dimensional nucleic acid structures. *Nucleic Acids Res* **31**, 5108-21 (2003).
3. Lu, X.J. & Olson, W.K. 3DNA: a versatile, integrated software system for the analysis, rebuilding and visualization of three-dimensional nucleic-acid structures. *Nat Protoc* **3**, 1213-27 (2008).
4. Lavery, R. & Sklenar, H. Defining the structure of irregular nucleic acids: conventions and principles. *J Biomol Struct Dyn* **6**, 655-67 (1989).
5. Lavery, R., Moakher, M., Maddocks, J.H., Petkeviciute, D. & Zakrzewska, K. Conformational analysis of nucleic acids revisited: Curves+. *Nucleic Acids Res* **37**, 5917-29 (2009).
6. Olson, W.K. et al. A standard reference frame for the description of nucleic acid base-pair geometry. *J Mol Biol* **313**, 229-37 (2001).
7. Reynolds, M.J., Hachicho, C., Carl, A.G., Gong, R. & Alushin, G.M. Bending forces and nucleotide state jointly regulate F-actin structure. *Nature* **611**, 380-386 (2022).
8. De La Cruz, E.M., Martiel, J.L. & Blanchoin, L. Mechanical heterogeneity favors fragmentation of strained actin filaments. *Biophys J* **108**, 2270-81 (2015).
9. Punjani, A., Rubinstein, J.L., Fleet, D.J. & Brubaker, M.A. cryoSPARC: algorithms for rapid unsupervised cryo-EM structure determination. *Nat Methods* **14**, 290-296 (2017).
10. Huehn, A. et al. The actin filament twist changes abruptly at boundaries between bare and cofilin-decorated segments. *J Biol Chem* **293**, 5377-5383 (2018).
11. Maggs, A.C. Writhing geometry at finite temperature: Random walks and geometric phases for stiff polymers. *The Journal of Chemical Physics* **114**, 5888-5896 (2001).
