# Supplementary material for: Cryo-EM structures reveal how phosphate release from Arp3 weakens actin filament branches formed by Arp2/3 complex
Source: Nat Commun. 2024 Mar 6;15:2059. doi: 10.1038/s41467-024-46179-x (PMC10918085; doi:10.1038/s41467-024-46179-x)
Supplement: Supplementary file 3 — Description of Additional Supplementary Files [file 41467_2024_46179_MOESM3_ESM.pdf]

File name: Supplementary Movie 1

Description: Animation of the structural comparison in Figure 5A, illustrating conformational changes of the ADP structure relative to the ADP-BeF_x structure. Morphing comparisons of the two structures are shown along with rigid-body extrapolated animations, where translations and rotations are amplified 5x. To perform the extrapolations, axes and amplitudes of subunit rotations and translations were obtained by the 'measure rotation' command of UCSF ChimeraX, following the method described in Fig. 3. The amplitudes (rotation angles and shifts) were then scaled up 5-fold. Fitted subunits/domains (ARPC2, ARPC3, ARPC4, ARPC5, ARPC6, Arp2 inner/outer domains and Arp3 inner/outer domains) from the ADP branch structure were then rotated/translated by these 5x amplitudes first in the forwards direction, and then the reverse. For this comparison, ADP and ADP-BeF_x structures were first aligned by least-squares superposition of the six mother filament subunits as in Fig. 5.

File name: Supplementary Movie 2

Description: Comparison of the ADP branch junction structure with a model in which Arp2 and Arp3 structures are substituted by fully un-flattened conformations seen in the GMF-Arp2/3 complex crystal structure [12]. The un-flattened Arp2 and Arp3 structures were aligned to the ADP branch structure by their inner and outer domains, respectively. This view is from the mother filament pointed end.
